# Effect of Botulinum Toxin on Masticatory Muscle Pain in Patients with Temporomandibular Disorders: A Randomized, Double-Blind, Placebo-Controlled Pilot Study

**DOI:** 10.3390/toxins15100597

**Published:** 2023-10-04

**Authors:** So Ra Kim, Min Chang, Alec Hyung Kim, Seong Taek Kim

**Affiliations:** 1 Department of Orofacial Pain and Oral Medicine, Yonsei University College of Dentistry, Seoul 03722, Republic of Korea; drsorakim@gmail.com (S.R.K.); minchang@yuhs.ac (M.C.); 2 TMJ & Orofacial Pain Center, Los Angeles, CA 90006, USA; asktmj@gmail.com

**Keywords:** botulinum toxin type A, masticatory muscle pain, temporomandibular disorders, headache

## Abstract

This study aimed to evaluate the efficacy of botulinum toxin type A (BoNT/A) in patients with temporomandibular disorders (TMDs) associated with masticatory muscle pain (MMP) and headaches. This randomized, double-blind, placebo-controlled pilot study is the first clinical trial to evaluate both disorders simultaneously. Twenty-one patients with myogenous TMD were randomly assigned to two groups. The experimental and control groups received injections of either BoNT/A or saline into the sites showing tenderness after palpation of a total of 16 muscle areas, including each masseter, a temporalis, splenius capitis, sternocleidomastoid, and trapezius muscle. During each visit, the clinical effects, based on the intensity of orofacial pain (OVAS), headache (HVAS), number of tender points (TPs), maximum mouth opening (MMO), and headache frequency (HF), were evaluated at four time points, namely, pre-injection and 4, 8, and 12 weeks after the injection, in both groups. Friedman and Mann–Whitney tests were used for the analyses. In the experimental group, the reductions in OVAS, TP, HVAS, and HF showed significant differences over time, excluding MMO, whereas there was no significant difference in any of the variables in the control group. In addition, the decline in TPs was significantly different between the experimental and control groups at all time points, especially after 4 and 12 weeks, compared to that during pre-injection. In conclusion, treatment with BoNT/A was relatively effective for masticatory muscle pain caused by TMDs and headache compared to the saline placebo.

## 1. Introduction

Temporomandibular disorders (TMDs) refer to a range of conditions affecting the masticatory muscles or the temporomandibular joint (TMJ) and are the second most common musculoskeletal disorders causing pain and functional impairment [1]. Patients with TMD may complain of pain in and around the joint, pain when opening and closing their mouths, difficulty in mastication and speech, and chronic headaches or earaches due to radiation of pain [2]. In addition, persistent pain and discomfort in the masticatory system can affect oral-health-related quality of life [3]. The diagnostic criteria include restricted jaw movement, problems relating to clicking sounds or locking in the TMJ, and discomfort worsened by jaw function [1].

TMDs are considered to have a multifactorial etiology, commonly associated with muscle hyperfunction, oral parafunction, traumatic injuries, hormonal influences, and articular changes within the joint [4]. Additionally, several studies have demonstrated an association between occlusal factors and TMJ symptoms, encompassing myogenous pain and functional impairments [5,6]. Occlusal stability ensures that it maintains optimal function of the masticatory system by adequately preserving the function of masticatory muscles [7]. Furthermore, a study indicated that patients with TMD muscle pain exhibit a higher frequency of oral dysfunctional activities, leading to greater electromyographic activity in the masticatory muscles compared to pain-free individuals [8]. Consequently, muscle overactivity can trigger the onset of masticatory muscle pain (MMP), characterized by persistent, dull pain or fatigue localized to the jaw and muscles around the TMJ [9].

It has been reported that approximately 50% of all TMDs comprise masticatory myalgia or painful masticatory muscle disorders [10]. MMP originates in the masticatory muscles, including the tendons and fascia, and is diagnosed when tenderness is present upon palpation of the masticatory muscle [11]. Patients with MMP in their frontalis or cervical muscles may experience headaches or cervical pain [12]. Headaches are one of the frequent symptoms among people with TMD. Ballegaard et al. [13] concluded that the prevalence of TMD among patients with headache was 56.1%, and about 46.9% of these TMD patients had MMP. This indicates that individuals with headache and TMD generally have several similar symptoms and diagnostic signs [12,13,14].

Since the US Food and Drug Administration first approved Botulinum toxin type A (BoNT/A) for the treatment of strabismus and blepharospasm in 1989, it has been approved for the treatment of hemifacial spasm, cervical dystonia, and hyperhidrosis and for cosmetic treatment [15,16]. BoNT inhibits the release of presynaptic acetylcholine at the neuromuscular junction, resulting in reduced postsynaptic muscle contraction [17]. It also has an antinociceptive effect, and its main mechanism of action is mediated through the blocking of neuropeptides and the release of inflammatory mediators [18]. Owing to its muscle activity reduction and pain-relief effects, BoNT/A is increasingly being used to treat muscle-related diseases associated with TMD, such as spasms and myofascial pain [19], with recent clinical reviews supporting its benefits in these treatments [20,21,22]. According to the American Academy of Neurology, through high-quality controlled clinical trials, injecting BoNT/A into TMD patients with myogenous pain was probably effective, with level B evidence [23]. In the guidelines of the American Academy of Neurology (updated in 2016), it was stated that, especially in terms of managing chronic migraine, the number of headache-free days can be increased, with level A evidence, and the effect of headache on health-related quality of life can be reduced, with level B evidence [24]. In addition, BoNT/A significantly reduces pain associated with a variety of conditions besides migraine headaches [25].

However, several double-blind randomized clinical trials (RCTs) failed to indicate significant differences with respect to myofascial pain in TMD between patients treated with BoNT/A and a saline placebo [26,27,28]. In addition, several studies have failed to demonstrate significant improvements in the treatment of headaches with BoNT/A compared to placebos [29,30,31,32]. Accordingly, there is no definitive evidence for its use in treating this disorder [33]. Heterogeneity of the results has been observed, prompting the need for additional RCTs to assess the efficacy of BoNT/A in establishing an effective treatment modality for TMD patients with muscle pain and headaches. Furthermore, there are several studies evaluating the efficacy of BoNT/A for TMD patients with pain related to masticatory muscles, such as the masseter muscle, temporal muscle, and lateral pterygoid muscles, or among headache patients, but there have not been any studies that evaluated these aspects simultaneously. The null hypothesis of this study posits that there is no therapeutic effect of BoNT/A compared to saline solution for TMD associated with MMP and headaches. The specific aim of this study is to verify this hypothesis and evaluate whether BoNT/A can be recommended as the optimal treatment for adult TMD patients with MMP and headaches.

## 2. Results

### 2.1. Participants

A total of 21 patients (19 women and 2 men; age range of 21–53 years) clinically diagnosed with MMP were included in the study. They were randomly divided into two groups and received BoNT/A or saline injections. There were no significant differences in sex and age between the groups; thus, the homogeneity of the general characteristics between the groups was confirmed. There were no dropouts from either group during the 12-week study period. In addition, there were no reports of side effects during this study. The participants’ demographic characteristics are presented in Table 1.

### 2.2. Differences between Groups

#### 2.2.1. Comparison of Changes over Time within Groups

**Figure 1:**
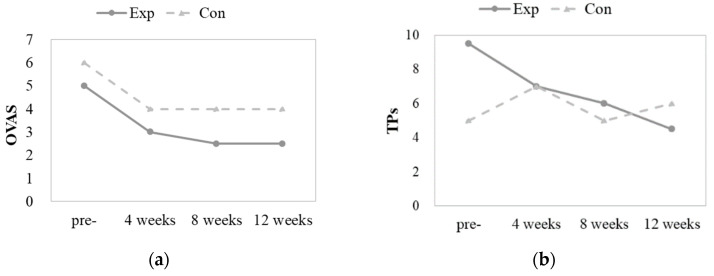

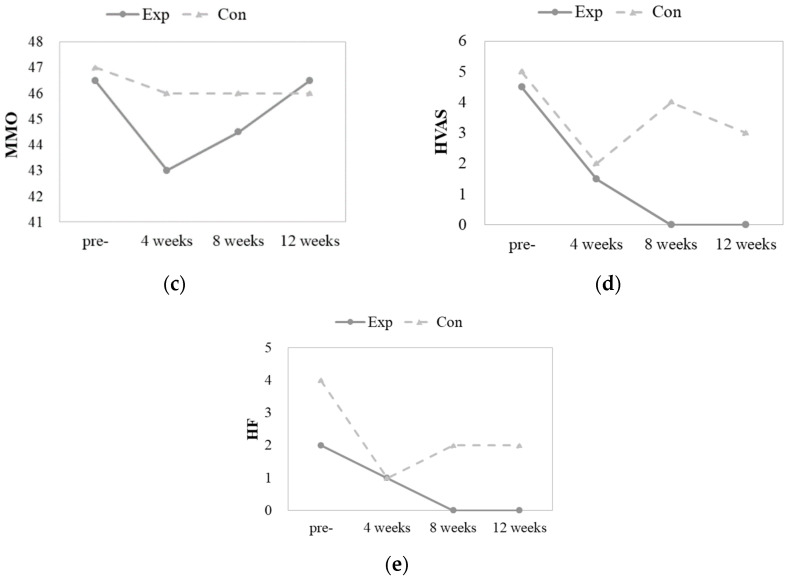
(**a**) OVAS change by group; (**b**) TP change by group; (**c**) MMO change by group; (**d**) HVAS change by group; (**e**) HF change by group. OVAS, the intensity of orofacial pain; TPs, the number of tender points; MMO, maximum mouth opening (mm); HVAS, the intensity of headache; HF, headache frequency per month; Exp, experimental group; Con, control group; pre-, pre-injection.

According to the Friedman test, which was used to verify whether the change over time was significant in the two groups, no significant difference in OVAS, TPs, MMO, HVAS, or HF was observed in the control group, whereas in the experimental group, except for MMO, OVAS (*p* = 0.003), TP (*p* < 0.001), HVAS (*p* = 0.005), and HF (*p* = 0.002) showed significant differences over time (Table 2). For the variables showing a significant difference, we verified whether they showed a significant difference between different time points using the Wilcoxon signed-rank test. OVAS was compared before injection (median = 5.00), after 4 weeks (median = 3.00), after 8 weeks (median = 2.50), and after 12 weeks (median = 2.50), and it was indicated that OVAS was significantly lower at all time points compared to that before injection. TPs values were significantly lower after 4 weeks (median = 7.00), 8 weeks (median = 6.00), and 12 weeks (median = 4.50) than they were before injection (median = 9.50). HVAS was also significantly lower after 4 weeks (median = 1.50), 8 weeks (median = 0.00), and 12 weeks (median = 0.00) than it was before injection (median = 4.50). In addition, HF was significantly lower after 8 (median = 0.00) and 12 weeks (median = 0.00) than that before injection (median = 2.00). Therefore, the OVAS, TPs, HVAS, and HF values significantly decreased in the experimental group but not in the control group (Figure 1).

#### 2.2.2. Comparison of Changes between Groups

A Mann–Whitney U test was conducted to verify whether there was a significant difference between the groups in the amount of change after 4, 8, and 12 weeks of pre-injection. Consequently, the changes in OVAS, MMO, HVAS, and HF did not show significant differences between the experimental and control groups at all time points. In contrast, TPs showed a significant difference between the groups in terms of the amount of change after 4 weeks (*p* = 0.009) and 12 weeks (*p* = 0.032) compared to that before injection. The amount of change after 4 weeks was greatly reduced in the experimental group (median = −3.00) compared to that in the control group (median = −1.00). Additionally, the amount of change after 12 weeks was greatly reduced in the experimental group (median = −5.00) compared to that in the control group (median = 1.00). These results indicate that the experimental group exhibited a greater reduction in TPs compared to the control group (Table 3). In addition, only TPs values in the experimental group showed a significant difference compared to those in the control group. Changes in other variables, such as OVAS, MMO, HVAS, and HF, did not show significant differences between the experimental and control groups at any time point (Table 3).

## 3. Discussion

Instead of conservative treatments, such as physical therapy, the use of non-steroidal analgesic agents, and the local injection of anesthetics and steroids, BoNT/A is being increasingly used as an adjuvant treatment for head and neck pain, such as tension-type headaches and migraine headaches [16]. BoNT/A, with enduring analgesic and muscle relaxant effects lasting around 3–4 months, has garnered significant interest as a potential therapy for myofascial pain and headache disorders [34]. Various studies have been conducted to establish BoNT as an effective treatment for myogenous pain among TMD patients. However, there are very few double-blind, randomized controlled pilot studies using a saline placebo, and even these yield inconsistent outcomes. Furthermore, there are no existing studies evaluating the effects of BoNT/A on both headaches and myogenous conditions among patients with TMDs at the same time. Thus, a demand has arisen for well-designed RCTs with new evidence-based methodologies evaluating the estimated effects of BoNT with enhanced reliability [35]. Consequently, a key emphasis of our study is the simultaneous assessment of the effects for each condition among patients experiencing both of these symptoms. This study aimed to test the hypothesis that BoNT injections are more efficacious than saline injection for treating TMD accompanied by MMP and headaches. As a result, the null hypothesis was partially rejected.

In this study, OVAS and TPs were compared to evaluate the change in the intensity of orofacial pain. Our findings showed a significant reduction in pain intensity over time in the experimental group compared to the control group, as demonstrated by a decrease in the OVAS over time and significant intergroup variation as well as time effects for TPs. Similarly, a double-blind RCT conducted by Lindern et al. [36] exhibited a statistically significant improvement of 3.2 points on the VAS in the analyzed BoNT/A injection group compared to a saline placebo group (*p* < 0.01). Furthermore, a selective crossover RCT conducted by Patel et al. [37] indicated decreased pain scores in the placebo group after one month (*p* = 0.01), while the BoNT/A group demonstrated a more substantial overall reduction (*p* = 0.0002) in pain scores, which remained statistically significant up to 2–4 months post-injection. Conversely, several studies demonstrating no significant difference in pain reduction between BoNT/A and saline injection groups also exist [26,27,38]. Nixdorf et al. [26] did not find a significant decrease in pain intensity or tender point count. However, this study had a small sample size and a high attrition rate, resulting in a high risk of attrition bias [39]. Similarly, Ernberg et al. [27] measured average pain intensity at baseline, 1 month, and 3 months, observing a significant time effect (*p* < 0.001), though no main difference between drugs was noted (2-way RM ANOVA, *p* = 0.163). Post hoc analysis indicated a significant decrease in pain intensity at 1 and 3 months. While the number of palpatory pain sites decreased after BoNT/A and saline injections, no statistically significant differences were found. Our study did not find a significant intergroup difference in OVAS, but significant differences were observed in TPs at 4 weeks (*p* = 0.009) and 12 weeks (*p* = 0.032) after BoNT/A injection compared to pre-injection. However, most of the studies mentioned above limited the scope of injection treatment to the masseter muscle or even included the temporalis. Nixdorf et al. [26] included the lateral pterygoid muscle, and Linder et al. [36] included the medial pterygoid muscle. Importantly, this study went beyond the usual scope of injection treatment, i.e., focusing solely on the masseter muscle or even the temporal muscle. Instead, we assessed pain in a broader range of muscles, including the splenius capitis, sternocleidomastoid muscle, and trapezius muscle. Moreover, injections were distributed bilaterally to pain areas, with a maximum of 150 units administered, allowing for a broader observable effect. Additionally, we used manual palpation, which is the most clinically appropriate method for evaluating tenderness in tissues surrounding the TMJ, including the masticatory systems [40]. In conclusion, our study underscores the significant therapeutic potential of reducing MMP.

Furthermore, in our study, no time effects for changes in MMO were observed in either group (exp; *p* = 0.189, con; *p* = 0.242), and there were no significant intergroup differences in MMO change. When comparing the MMO values of all the participants, it is clear that there were no restrictions of mouth opening in both the experimental groups (median = 46.50) and control groups (median = 47.00). Thus, a substantial improvement in post-treatment mandibular range of motion was not anticipated. However, clinical limitations in mandibular range of motion due to masticatory muscle pain may occur, resulting in a reduction in range of motion [41]. In such cases, BoNT/A treatment can facilitate the relaxation of adjacent masticatory muscles, mitigate muscle inflammation, and improve mouth opening [39,42]. Therefore, mandibular range of motion can serve as a parameter for evaluating the therapeutic efficacy of BoNT/A. There are available studies showing a significant enhancement in MMO range following BoNT injections. De la Torre Canales et al. [35] recently reported significant MMO improvement in their analyzed BoNT/A group compared to a saline injection group after 180 days, regardless of the administered dose (*p* < 0.05), and a significant improvement was observed in comparisons between groups (*p* < 0.05). Guarda-Nardini et al. [28] similarly reported a significant MMO increase over time in their BoNT/A group compared to a saline placebo group at baseline and at 1 week, 1 month, and 6 months post-injection. However, similar to our study, Ernberg et al. [27] evaluated changes in unassisted pain-free jaw opening and did not find any significant differences in either group. Remarkably, Nixdorf et al. [26] noted a significant increase in MMO after 3 months of injection in a saline placebo group, while the BoNT group showed a reduction in MMO. Consequently, a definitive conclusion about whether BoNT injections improve mandibular range of motion remains elusive. Therefore, conducting pilot studies with larger sample sizes seems necessary.

In addition, we investigated the effect of BoNT treatment on headache by assessing HVAS and HF. Both HVAS (*p* = 0.005) and HF (*p* = 0.002) demonstrated time effects in the experimental group. Instances of headache resolution at 4 and 8 weeks post-injection were also observed. However, the experimental group generally displayed a slight decrease in median values when comparing the changes at 4, 8, and 12 weeks to the pre-injection levels, though these changes were statistically insignificant. Headaches are classified into primary and secondary headaches, according to the third version of the International Classification of Headache Disorders (ICHD-3). Headaches also occur in association with TMD and can be identified according to the diagnostic criteria for Diagnostic Criteria for TMDs (DC/TMD) [1]. In cases where such headaches have a muscular origin, BoNT/A is suggested as an attractive option for the treatment of TMD and headaches given its potential to relieve muscular hyperactivity [2]. BoNT/A has proven to be effective for pain relief for various headache disorders, including masticatory muscle pain, tension headache, chronic migraine, and chronic daily headache [43,44]. However, several studies with inconclusive statistical significance have also been presented, leading to ongoing debates about its efficacy [26,27,28,29,30,31,32,33]. In our study, the participants were clinically diagnosed with general headache based on their pain history and clinical examination. The reported headache frequency and pain intensity values were reduced in this study, and these results were partially statistically significant. Nonetheless, it is difficult to completely explain the effectiveness of BoNT/A for specific headaches only with the results of this study. Therefore, further well-designed trials are imperative, aiming to classify cases under well-defined diagnostic criteria, specify specific headache diagnoses, and meticulously evaluate the precise clinical effects of BoNT/A. These efforts are essential for proposing optimized therapeutic approaches.

As mentioned above, several studies have only mentioned the pain relief effect of BoNT/A on the masticatory muscles of patients with TMD; however, there has been a lack of prospective, randomized, double-blind approaches to assessing the effect of BoNT/A on both MMP and headache among patients with TMD. Therefore, our study aimed to simultaneously evaluate the effect of BoNT/A in treating both MMP and headache, and the following factors were analyzed: OVAS, TPs, MMO, HVAS, and HF.

This study has several limitations. The major limitation of this study was its small sample size. Second, this study was carried out on patients with general headaches without classifying the headaches into detailed diagnoses, such as migraine or tension-type headaches. Despite these limitations, this is the first prospective study to evaluate the therapeutic effects of BoNT/A on both MMP and headache among patients with TMD. Moreover, we adopted a double-blind RCT design to collect evidence-based data on the use of BoNT/A among TMD patients with MMP and headache, enhancing data reliability. Considering the outcomes derived from this pilot study, further studies with larger sample sizes are warranted. Furthermore, it is necessary to conduct additional RCTs and prospective studies to distinctly classify headache diagnoses according to the criteria of the Headache Classification Committee of the International Headache Society. This approach will ultimately allow for a more optimized evaluation of BoNT’s clinical effects on headaches.

## 4. Conclusions

According to our study results, after BoNT/A injection, significant values were observed in the experimental group over time, and TPs decreased. Therefore, BoNT/A injection can be suggested as a relatively effective treatment for patients with TMD presenting with MMP and headache compared to saline placebo.

## 5. Materials and Methods

### 5.1. Subjects

The clinical trial included 21 patients (2 men and 19 women; age range of 21–53 years) with chief complaints of MMP with TMDs and headache who were enrolled at the Department of Orofacial Pain and Medicine, Dental University of Yonsei, Seoul, Republic of Korea. At their first visit, patients were screened to determine whether they qualified to participate in the trial, and clinical values were measured prior to injection. To exclude the patients with arthralgia, diagnostic criteria (DC/TMD) were applied, and the following clinical criteria for TMD were satisfied: (1) history of pain in the masticatory muscle reported for ≥5 days in the previous 30 days; (2) pain induced by the clinician’s palpation of the masticatory muscles (total of 16 sites; 2 points in the masseter muscle and 3 points in the temporal muscle, and 1 point in the splenius capitis, sternocleidomastoid muscle, and trapezius muscle, each assessed bilaterally). The exclusion criteria were as follows: (1) patients whose pain was caused by a disc or joint-related disease; (2) patients with a history of muscle-related diseases that affect neuromuscular function, such as myasthenia gravis, Eaton–Lambert syndrome, amyotrophic lateral sclerosis, and motor neuropathy; (3) patients who had received an injection of BoNT within a year from the study or had been administered a myofascial trigger point injection, such as lidocaine, procaine, or bupivacaine, within a month from the study based on screening; (4) patients diagnosed with or treated for fibromyalgia; and (5) women who were pregnant, lactating, or of childbearing potential. All participants understood the content of the study and voluntarily signed a consent form. This study was approved by the Institutional Review Board of the Yonsei University Dental Hospital (IRB No. 2-2019-0061) on 14 November 2019.

### 5.2. Study Design

This was a prospective, randomized, double-blind, placebo-controlled clinical trial. Clinicians checked the demographic information, medical history, and medication history of participants. Participants completed questionnaires regarding TMD and headache pain for measurement and assessment of clinical values. The intensity of orofacial pain was assessed using a VAS, and participants indicated the frequency and intensity of various types of headaches experienced during the past 30 days. Additionally, the clinicians measured the number of tender points and the range of maximum mouth opening in millimeters (mm). Accordingly, diagnoses were clinically determined. Participants who met the selection criteria and did not meet the exclusion criteria were randomly assigned to either the test group (*n* = 14; treated with BoNT/A injections) or the control group (*n* = 7; treated with saline placebo injections). The injection was administered in the area where there was tenderness during palpation. Different amounts of BoNT/A (experimental group) or saline (control group) were administered depending on the muscle. A total of 100 U of BoNT/A (Botulax^®^, Hugel Inc., Chuncheon, Republic of Korea) was supplied in a freeze-dried powder state and reconstituted to a concentration of 10 U/0.2 mL using 2 mL of sterile saline solution. A 1 mL syringe with a 29-gauge, 0.5-inch needle was used. A total of 10 U of BoNT/A was injected into the muscle area at which patients complained of pain among the 16 designated tender points, ensuring that the total dose did not exceed 150 U. The participants visited our department on the day of injection and at 4, 8, and 12 weeks post-injection. At each visit, the clinical effects were measured and compared, including OVAS, MMO, HVAS, HF, and TPs. Participants recorded OVAS and, if headaches persisted, indicated the HF and HVAS they had experienced during the past one month.

### 5.3. Statistical Analysis

The normality of the data distribution was examined using the Shapiro–Wilk test. As a result, some variables satisfied the assumption of normality, but several variables did not; therefore, we decided to test the hypothesis using non-parametric statistics. Descriptive analyses were performed using the mean and standard deviation for normally distributed variables and median and interquartile range values for non-normally distributed and ordinal variables. The chi-square test (χ^2^ test) and independent samples *t*-test were conducted to verify the homogeneity of the general characteristics between the groups. The Mann–Whitney U test was conducted to determine the prior homogeneity of the major variables between the groups. We verified whether there was a significant difference between the groups in the amount of change after 4, 8, and 12 weeks from baseline. The Friedman test was performed to verify the significance of the changes over time in the experimental and control groups. The Mann–Whitney U test was used to compare the amount of change between the experimental and control groups. Statistical analysis was performed using IBM SPSS statistics version 26.0 (IBM Corp., Armonk, NY, USA) at a significance level of *p* < 0.05.

## Figures and Tables

**Table 1:** Homogeneity of general characteristics between the experimental and control groups.

Variables	Exp (*n* = 14)	Con (*n* = 7)	Total (*n* = 21)	*p*-Value
Sex				
Male	2(14.3)	0(0.0)	2(9.5)	0.293
Female	12(85.7)	7(100.0)	19(90.5)	
Age (M ± SD)	34.71 ± 9.73	32.43 ± 10.23	33.95 ± 9.70	0.623

Exp, experimental group; Con, control group.

**Table 2:** Differences in variables in the experimental and control groups according to time.

Variables	Group	Time	M ± SD	Q1	Median	Q3	*p*-Value
OVAS	Exp	Pre-injection	5.00 ± 1.45	4.00	5.00 ^a^	6.13	0.003 *
		4 weeks	2.64 ± 2.02	0.00	3.00 ^b^	4.25	
		8 weeks	2.39 ± 2.24	0.00	2.50 ^b^	4.00	
		12 weeks	2.50 ± 2.31	0.00	2.50 ^b^	4.25	
	Con	Pre-injection	5.36 ± 1.44	4.00	6.00	6.50	0.256
		4 weeks	3.64 ± 2.06	2.00	4.00	5.00	
		8 weeks	3.79 ± 2.00	3.00	4.00	5.00	
		12 weeks	3.43 ± 1.81	3.00	4.00	4.00	
TPs	Exp	Pre-injection	10.07 ± 3.79	7.50	9.50 ^a^	12.75	<0.001 *
		4 weeks	6.86 ± 3.61	3.00	7.00 ^b^	10.25	
		8 weeks	5.86 ± 4.66	2.50	6.00 ^b^	8.50	
		12 weeks	5.07 ± 4.03	1.50	4.50 ^b^	9.25	
	Con	Pre-injection	7.43 ± 4.54	4.00	5.00	10.00	0.588
		4 weeks	6.86 ± 4.06	4.00	7.00	7.00	
		8 weeks	5.57 ± 3.21	4.00	5.00	8.00	
		12 weeks	5.86 ± 4.60	1.00	6.00	11.00	
MMO	Exp	Pre-injection	46.57 ± 7.18	41.75	46.50	50.25	0.189
		4 weeks	45.71 ± 7.96	40.00	43.00	50.25	
		8 weeks	46.57 ± 7.97	40.75	44.50	52.75	
		12 weeks	47.21 ± 8.09	40.75	46.50	52.75	
	Con	Pre-injection	48.00 ± 6.61	42.00	47.00	52.00	0.242
		4 weeks	47.00 ± 5.72	42.00	46.00	50.00	
		8 weeks	46.29 ± 5.02	41.00	46.00	50.00	
		12 weeks	46.43 ± 3.95	43.00	46.00	50.00	
HVAS	Exp	Pre-injection	3.61 ± 2.63	0.00	4.50 ^a^	6.00	0.005 *
		4 weeks	2.00 ± 2.18	0.00	1.50 ^b^	4.00	
		8 weeks	1.64 ± 2.10	0.00	0.00 ^b^	4.00	
		12 weeks	1.46 ± 2.45	0.00	0.00 ^b^	4.13	
	Con	Pre-injection	4.79 ± 1.15	4.00	5.00	5.00	0.768
		4 weeks	2.36 ± 2.46	0.00	2.00	5.00	
		8 weeks	3.36 ± 2.63	0.00	4.00	5.50	
		12 weeks	3.00 ± 1.94	1.00	3.00	4.50	
HF	Exp	Pre-injection	5.71 ± 8.43	0.00	2.00 ^a^	12.00	0.002 *
		4 weeks	2.00 ± 3.92	0.00	1.00 ^ab^	2.00	
		8 weeks	1.29 ± 2.64	0.00	0.00 ^b^	2.00	
		12 weeks	1.36 ± 2.53	0.00	0.00 ^b^	2.50	
	Con	Pre-injection	8.00 ± 10.15	2.00	4.00	10.00	0.119
		4 weeks	5.43 ± 10.95	0.00	1.00	4.00	
		8 weeks	2.71 ± 3.45	0.00	2.00	3.00	
		12 weeks	1.93 ± 1.17	1.00	2.00	3.00	

Exp, experimental group; Con, control group; OVAS, the intensity of orofacial pain; TPs, the number of tender points; MMO, maximum mouth opening (mm); HVAS, the intensity of headache; HF, headache frequency per month. An asterisk (*) indicates statistical significance (*p* < 0.05). Different letters indicate significant differences.

**Table 3:** Differences in changes in variables according to group.

Variables	Time	Group	M ± SD	Q1	Median	Q3	*p*-Value
OVAS	4 weeks—pre-injection	Exp	−2.36 ± 2.38	−4.00	−2.75	0.00	0.474
		Con	−1.71 ± 2.69	−3.00	−1.00	0.00	
	8 weeks—pre-injection	Exp	−2.61 ± 2.28	−4.00	−2.75	0.00	0.244
		Con	−1.57 ± 2.71	−2.50	−1.00	0.50	
	12 weeks—pre-injection	Exp	−2.50 ± 2.45	−4.00	−2.00	−1.00	0.430
		Con	−1.93 ± 2.62	−3.00	−1.00	0.00	
TPs	4 weeks—pre-injection	Exp	−3.21 ± 1.58	−4.25	−3.00	−2.00	0.009 *
		Con	−0.57 ± 2.23	−2.00	−1.00	2.00	
	8 weeks—pre-injection	Exp	−4.21 ± 3.17	−6.00	−4.00	−2.00	0.076
		Con	−1.86 ± 3.53	−4.00	0.00	0.00	
	12 weeks—pre-injection	Exp	−5.00 ± 2.15	−6.00	−5.00	−3.75	0.032 *
		Con	−1.57 ± 5.32	−4.00	1.00	2.00	
MMO	4 weeks—pre-injection	Exp	−0.86 ± 1.99	−3.00	0.00	0.00	0.696
		Con	−1.00 ± 1.15	−2.00	−1.00	0.00	
	8 weeks—pre-injection	Exp	0.00 ± 2.45	−2.25	0.00	1.25	0.121
		Con	−1.71 ± 2.36	−5.00	−1.00	0.00	
	12 weeks—pre-injection	Exp	0.64 ± 2.84	−1.25	0.00	3.00	0.240
		Con	−1.57 ± 3.15	−5.00	0.00	1.00	
HVAS	4 weeks—pre-injection	Exp	−1.61 ± 2.42	−2.63	−0.50	0.00	0.647
		Con	−2.43 ± 2.95	−4.00	−3.00	0.50	
	8 weeks—pre-injection	Exp	−1.96 ± 2.34	−3.25	−1.50	0.00	0.377
		Con	−1.43 ± 2.28	−4.00	0.00	0.50	
	12 weeks—pre-injection	Exp	−2.14 ± 2.26	−4.25	−2.00	0.00	0.548
		Con	−1.79 ± 2.53	−4.00	−2.00	0.50	
HF	4 weeks—pre-injection	Exp	−3.71 ± 8.52	−4.00	0.00	0.00	0.489
		Con	−2.57 ± 3.26	−6.00	−2.00	0.00	
	8 weeks—pre-injection	Exp	−4.43 ± 8.30	−5.50	−1.00	0.00	0.493
		Con	−5.29 ± 7.02	−7.00	−4.00	0.00	
	12 weeks—pre-injection	Exp	−4.36 ± 7.22	−6.00	−1.50	0.00	0.567
		Con	−6.07 ± 9.44	−8.00	−3.00	0.00	

Exp, experimental group; Con, control group; OVAS, the intensity of orofacial pain; TPs, the number of tender points; MMO, maximum mouth opening (mm); HVAS, the intensity of headache; HF, headache frequency per month; An asterisk (*) indicates statistical significance (*p* < 0.05).

## Data Availability

The data presented in this study are available in this article.
